# A Simple Model Established by Blood Markers Predicting Overall Survival After Radical Resection of Pancreatic Ductal Adenocarcinoma

**DOI:** 10.3389/fonc.2020.00583

**Published:** 2020-04-30

**Authors:** Li-xiang Zhang, Lei Chen, A-Man Xu

**Affiliations:** ^1^Department of Gastrointestinal Surgery, Department of General Surgery, The First Affiliated Hospital of Anhui Medical University, Hefei, China; ^2^Department of General Surgery, Xiang'an Hospital Affiliated to Xiamen University, Xiamen, China; ^3^Department of General Surgery, The First Affiliated Hospital of Wenzhou Medical University, Hefei, China

**Keywords:** pancreatic ductal adenocarcinoma, NLR, LMR, prognosis, cancer

## Abstract

**Background:** The prognostic prediction after radical resection of pancreatic ductal adenocarcinoma (PDAC) has not been well-established. We aimed to establish a prognostic model for PDAC based on a new score system, which included a clinical routine serum marker.

**Methods:** A total of 438 patients who underwent curative PDAC at the First Affiliated Hospital of Anhui Medical University from January 2007 to January 2014 were included in this study. Univariate and multivariate analyses were used to screen for prognostic risk factors. We constructed the nomogram based on Cox proportional hazard regression models. The construction of the new score models was analyzed by the receiver operator characteristic curve (ROC curve), which were compared with other clinical indexes.

**Results:** Multivariate analysis showed that TNM stage, CA199, CEA, globulin, neutrophil-to-lymphocyte ratio (NLR), and lymphocyte-to-monocyte ratio (LMR) were independent prognostic factors. The new score system had a higher AUC value than other risk factors, and the C-index of the nomogram was highly consistent for evaluating survival of PDAC patients in the validation groups and training group, and the external population also verified the nomogram.

**Conclusions:** For the patients with PDAC after radical surgery, we developed a precise model to predict the prognosis based on the serum markers and other clinical indicators. For surgeons and patients, this score system can be an effective help.

## Introduction

Pancreatic cancer mortality ranks fifth in the most common cancers (1), and pancreatic ductal adenocarcinoma (PDAC) accounts for more than 90% of all pancreatic cancer cases (2). Pancreatectomy is considered the only means of curative treatment of PDAC, which provides a chance of cure and longer survival; however, the cancer recurrence rate and prognosis are still not optimistic even after radical resection, and the 5-year survival rate is only 25% (3). TNM stage, tumor size, vascular invasion, and other tumor pathological characteristics are associated with the prognosis of PDAC; however, they are hard to get before surgery. At present, studies indicated that serum markers correlated with the cancer-specific survival time (4–6); among them, neutrophil-to-lymphocyte ratio (NLR), platelet-to-lymphocyte ratio (PLR), and lymphocyte-to-monocyte ratio (LMR) as predictors have been studied worldwide in recent years. Additionally, there are also other serum indexes which can evaluate the prognosis of cancer. Aspartate aminotransferase (AST)/alanine aminotransferase (ALT) was reported to be the predictor of overall survival (OS) in some tumors (7). Besides, the level of albumin and the albumin globulin ratio, which can reflect the nutrient condition, are associated with the prognosis of patients (8). In this study, we are trying to find more clinical serum markers which can help assess the prognosis of PDAC and then build a reliable new score system.

Cancer-related systemic inflammatory response pushes forward an immense influence on the progression and outcome of tumors (4, 9), such as NLR, PLR, and LMR. Studies also found blood enzyme markers including γ-glutamyl transpeptidase (GGT), AST, and ALT, which can reflect the liver function and play a vital clinical significance in the prognosis of some cancers (10, 11). The presence of malnutrition can cause postoperative complications and poor prognosis (12), and nutritional markers have significant value for predicting survival. However, the relationship between these blood indexes and prognosis after PDAC resection remains unclear.

There are few studies that had republished to access the prediction of inflammatory markers, nutritional markers, and blood enzyme markers for OS in PDAC patients. Nomogram is a statistic model with a high reliability. In this research, we established a nomogram to explore the value of blood markers and then built a reliable model to predict OS after radical resection of PDAC.

## Patients and Methods

### Patients

We collected blood data and clinical data from PDAC patients who were hospitalized in the First Affiliated Hospital of Anhui Medical University from January 2007 to January 2014. According to the inclusion and exclusion criteria, patients were analyzed retrospectively during the research. The major criteria for inclusion in the study were included: (1) all patients were diagnosed as PDAC by pathological diagnosis; (2) the tumor is resectable; (3) the patients did not have heart disease or any major organ failure; and (4) peripheral blood tests were obtained within 1 week before operation. The major exclusion criteria for this study are as follows: (1) patients have a history of malignant tumors or various primary tumors; (2) they had received radiotherapy or chemotherapy before treatment; (3) they suffered from certain diseases that might affect peripheral blood cell count, such as infection; and (4) patients died because of lung infection, pulmonary embolism, and other surgery complications. This study included 438 PDAC patients and the external population including 82 PDAC patients who were hospitalized in the First Affiliated Hospital of Wenzhou Medical University from January 2015 to January 2016.

### Data Collection and Follow-Up

Through the hospital medical record room, we collected the patient's demographic data and clinical pathology data, including age, gender, tumor location, tumor size, differentiation grade, vascular invasion, and nerve invasion.

According to the AJCC 7th TNM staging system, we categorized the pathological tumor stage. The routine laboratory data for testing were as follows: neutrophil, lymphocyte, platelet, ALT, AST, GGT, albumin, globulin, and so on.

We obtained the peripheral blood tests within a week before operation, and the indexes NLR, PLR, and LMR were determined. NLR was counted by strict neutrophil count divided by strict lymphocyte count. PLR was counted by strict platelet count divided by strict lymphocyte count. LMR was counted by dividing the strict lymphocyte count by the strict monocyte count. According to the median, all variables (CEA, CA199, AFP, NLR, PLR, LMR, etc.) were divided into a low group and a high group.

The enrolled patients were followed up as expected. Their follow-up dates were obtained by telephone and from the clinic. The behavior was performed at regular intervals (every 90 days in 2 years after surgery, every 180 days in 3 or 5 years of age, and every year after 5 years). We followed up all patients, excluding 74 patients, of whom 54 lost contact, 12 died of non-cancer-related deaths, and 8 died because of surgical complications after surgery, and finally, 438 PDAC patients were included in the study. And we also got the 1- and 3-year survival information of the external population.

### Statistical Analysis

Continuous variables were expressed as mean ± standard deviation, and they were analyzed by Student *T*-test; categorical values were identified by quartile (P25, P75), and they were counted by chi-square test or Fisher exact test. The multivariate and univariate survival analyses were carried out using the Cox appropriate hazard pattern. We used the Harrell concordance index (C-index) in the nomogram to assess the model performance. The accuracy of the new scoring system was verified by the receiver operating characteristic (ROC) curve and the calibration curve. The SPSS app (version 16.0) and RStudio software (1.1.447-2009-2018; RStudio, Inc.) were used to described the entire data.

## Results

### Baseline Characteristics

The baseline characteristics of 438 patients (306 in the training group and 132 in the validation group) showed no significant difference in most variables between the training group and the validation group (*p* > 0.05) (Table 1).

**Table 1:** Baseline demographics and clinical characteristics of patients in training cohort and validation cohort.

**Variables**	**Training cohort(*n* = 306)**	**Validation cohort(*n* = 132)**	***P***
Age (years)	56.2 ± 11.4	55.9 ± 11.0	0.751
Body mass index (Kg/m^2^)	22.4 (20.6, 24.5)	23.0 (20.9,24.5)	0.249
Tumor size (cm)	4.0 (3.0,6.0)	4.0 (3.0,5.0)	0.561
Prothrombin time activity(s)	13.3 (12.7, 13.9)	13.20 (12.60,13.83)	0.399
APTT(s)	36.90 (34.32,40.2)	36.40 (34.0,40.3)	0.291
ALT	45 (19,203)	39 (18,140.5)	0.177
AST	36 (18, 95)	42 (19,133.25)	0.135
GGT	75 (20.0,420.0)	86.5 (20.0, 573.5)	0.200
CEA (g/L)	3.0 (1.6,5.65)	3.85 (2.1, 7.12)	0.986
CA199 (μmol/L)	74 (10, 717.02)	136.9 (19, 1019.75)	0.236
AFP (mmol/l)	2.80 (2.0, 4.0)	2.98 (2.35, 4.03)	0.301
Gender			0.836
Male	170 (55.56%)	92 (69.70%)	
Female	136 (44.44%)	40 (30.30%)	
T stage			0.001
T1	20 (6.54%)	13 (9.85%)	
T2	143 (46.73%)	35 (26.52%)	
T3	126 (41.18%)	71 (53.79%)	
T4	17 (5.56%)	13 (9.85%)	
N stage			0.004
N0	209 (68.30%)	71 (53.79%)	
N1-3	97 (31.70%)	61 (46.21%)	
M stage			0.011
M0	266 (86.93%)	102 (77.27%)	
M1	40 (13.07%)	30 (22.73%)	
Nerve invasion			0.014
Yes	248 (81.05%)	93 (70.45%)	
No	58 (18.95%)	39 (29.55%)	
Vascular invasion			0.067
Yes	230 (75.16%)	88 (66.67%)	
No	76 (24.84%)	44 (33.33%)	

*BMI, Body mass index; AFP, Alpha-fetoprotein; ALT, Alanine aminotransferase; AST, Aspartate aminotransferase; GGT, γ-glutamyl transpeptidase*.

### Prognostic Factors of the Training Cohort

Univariate risk factors of OS are shown in Table 2. The result showed that albumin, globulin, CEA, CA199, LMR, PLR, NLR, vascular invasion, nerve invasion, TNM, tumor size, and GGT were significant indicators, and *p*-values of variables less than 0.05 in univariate analysis were included in the multivariate analysis. The results showed that TNM, CEA, CA199, LMR, NLR, and globulin were independent prognostic factors for OS (Table 3).

**Table 2:** Univariate analysis of the training cohort.

**Variable**	**β**	**HR (95% CI for HR)**	***P***
**Statistically non-significant factors**			
Gender	−0.478	0.620 (0.330–1.165)	0.137
Age	0.378	1.460 (0.909–2.346)	0.118
BMI	0.001	1.001 (0.999–1.001)	0.057
ALT	0.001	1.001 (0.999–1.001)	0.075
Hemoglobin	0.099	0.905 (0.717–1.141)	0.401
APTT	0.011	1.011 (0.984–1.038)	0.413
Triglycerides	0.017	1.016 (0.806–1.281)	0.888
Cholesterol	−0.076	0.926 (0.735–1.167)	0.518
AFP	0.043	0.957 (0.759–1.206)	0.710
Location(head and other location)	0.038	1.038 (0.814–1.324)	0.759
**Statistically significant factors**			
Tumor size	0.396	0.673 (0.502–0.903)	0.008
NLR	0.754	2.124 (1.675–2.695)	<0.001
PLR	0.463	1.588 (1.257–2.004)	<0.001
LMR	−0.782	0.457 (0.360–0.579)	<0.001
CEA	0.500	1.650 (1.305–2.085)	<0.001
CA199	0.562	1.754 (1.390–2.215)	<0.001
γ-GT	0.001	1.000 (0.999–1.000)	0.250
TNM stage(I/II vs III/IV)	0.657	1.928 (1.468–2.532)	<0.001
Nerve invasion	0.323	1.380 (1.057–1.802)	0.017
Vascular invasion	0.409	1.505 (1.173–1.932)	0.001
Globulin	0.339	0.7125 (0.563–0.901)	<0.001
Albumin	−0.245	0.782 (0.620–0.986)	0.038
AST	0.001	1.001 (1.000–1.002)	0.024
Differentiation grade	0.104	1.109 (1.009–1.218)	0.030
PT	0.103	1.108 (1.001–1.227)	0.047

**Table 3:** Multivariate analysis of the training cohort.

**Variable**	**Exp(coef)**	**Exp(–coef)**	**Lower 0.95**	**Upper 0.95**	***P***
LMR	0.6821	1.4660	0.5015	0.9278	0.01478
CA199	1.4253	0.7016	1.1165	1.8195	0.00444
CEA	1.3577	0.7366	1.0614	1.7366	0.01492
TNM	1.5900	0.6289	1.1934	2.1185	0.00154
NLR	1.5422	0.6484	1.1416	2.0833	0.00476
Globulin	0.7810	1.2805	0.6158	0.9903	0.04135

### Prognostic Nomogram for Survival

**Figure 1:**
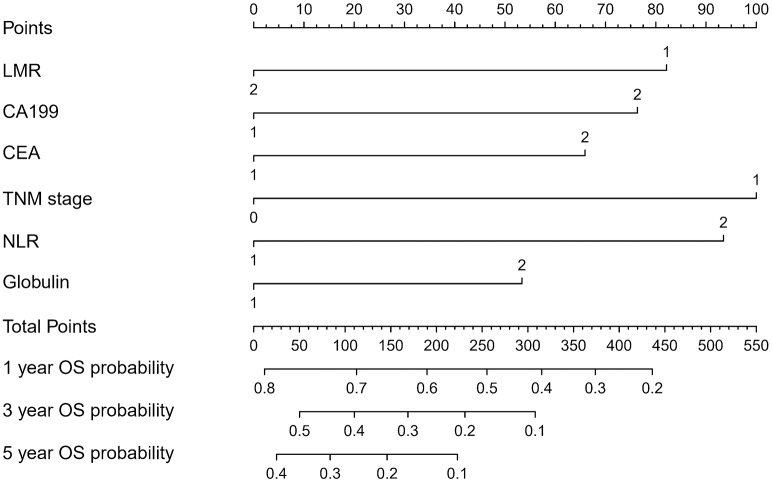
Nomogram for predicting overall survival after curative resection of PDAC.

The OS rate of PDAC was predicted by constructing a nomogram based on COX regression models (Figure 1). Each subgroup variable was assigned a corresponding score for the construction of this nomogram. A score system was used to assign a score of 0 to 100 for each subgroup variable according to the specific situation of each PDAC, and then we predicted the corresponding OS rates. The nomogram scoring system is shown in Table 4.

**Table 4:** Nomogram scoring system.

**LMR**	**Points**	**NLR**	**Points**	**CA199**	**Points**	**CEA**	**Points**	**TNM**	**Points**	**Globulin**	**Points**
High	0	High	93	Low	0	Low	0	I,II	0	Low	0
Low	82	Yes	0	High	76	High	66	III,IV	100	High	53
**Total points**		**1-Year survival probability**		**Total points**		**3-year survival probability**		**Total points**		**5-year survival probability**	
436		0.2		308		0.1		223		0.1	
374		0.3		231		0.2		146		0.2	
315		0.4		169		0.3		84		0.3	
255		0.5		110		0.4		25		0.4	
190		0.6		50		0.5					
113		0.7									
12		0.8									

### Validation of the Nomogram

**Figure 2:**
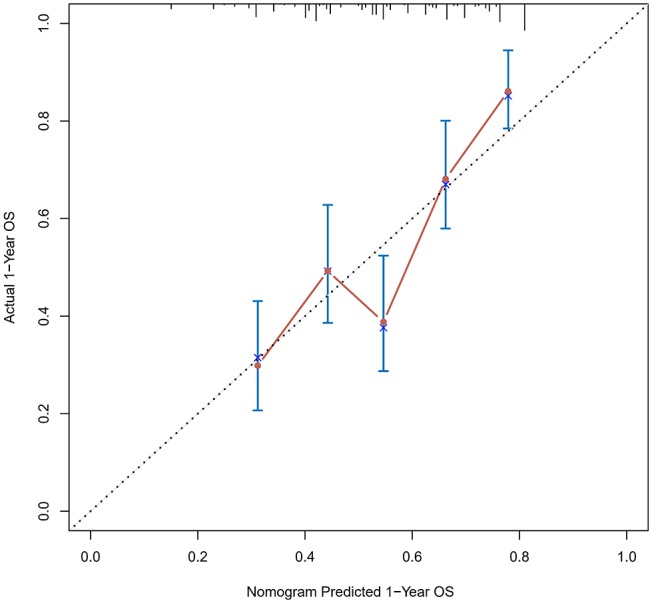
Calibration curves of the prognostic nomogram for 1-year overall survival in the training set.

**Figure 3:**
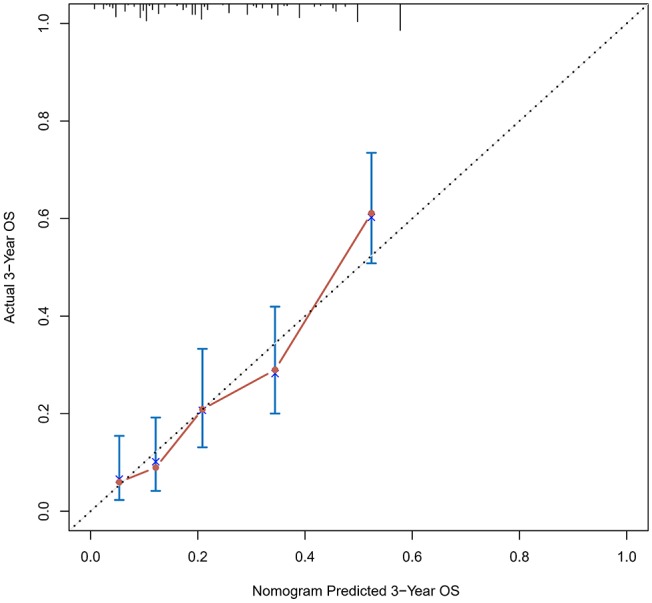
Calibration curves of the prognostic nomogram for 3-year overall survival in the training set.

**Figure 4:**
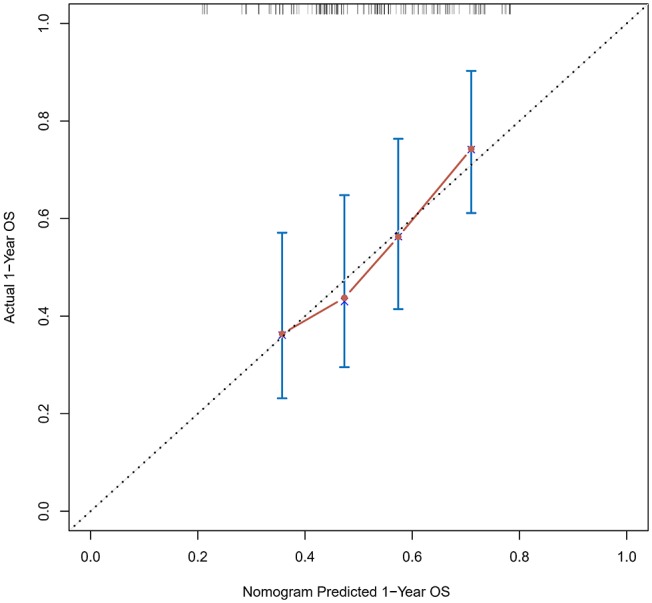
Calibration curves of the prognostic nomogram for 1-year overall survival in the validation set.

**Figure 5:**
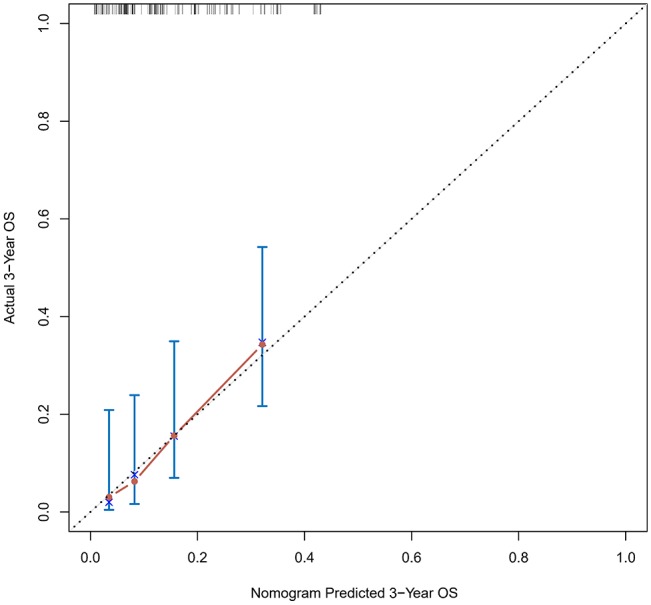
Calibration curves of the prognostic nomogram for 3-year overall survival in the validation set.

**Figure 6:**
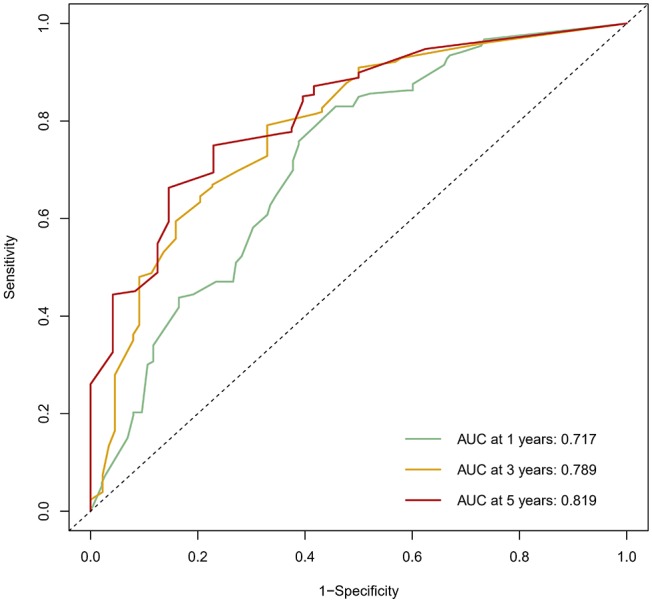
The ROC curve of the prognostic nomogram in the training set.

**Figure 7:**
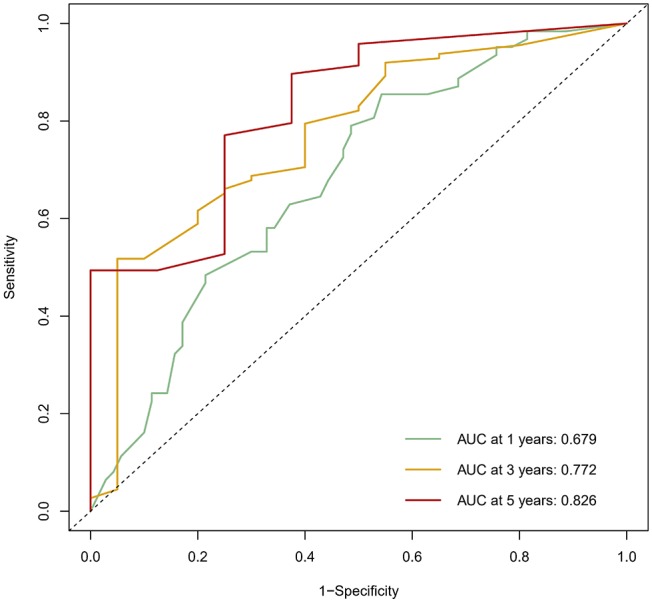
The ROC curve of the prognostic nomogram in the validation set.

The calibration curve was used to validate the model's ability for predicting the OS of patients with PDAC (Figures 2–5). The model C-index values in the training group and the verification group were 0.697 and 0.634, respectively. To further validate the performance of the model, the ROC curve was plotted for the nomogram (Figures 6, 7), and the area under the curve (AUC) of the nomogram was large, which indicated that the constructed nomogram was a reliable score system.

### Kaplan–Meier Curves

**Figure 8:**
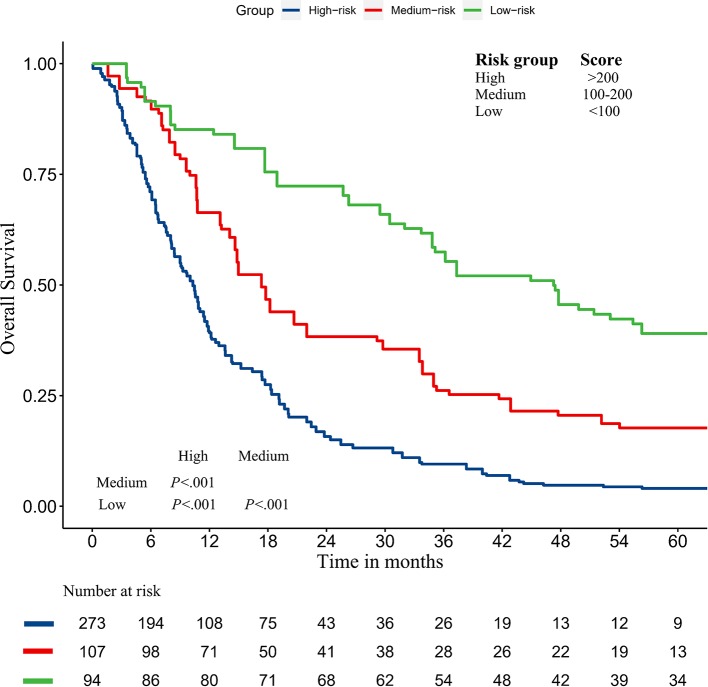
Survival curves stratified by the score calculated by the nomogram in the training cohort (low risk: <100; intermediate risk: 100–200; and high risk: >200).

In addition, we divided the training group into three groups according to the total score of the nomogram (low risk: <100; intermediate risk: 100–200; and high risk: >200) (Figure 8). The Kaplan–Meier curves show excellent prediction results in the nomogram predicting survival.

### Verification Through the External Population

**Figure 9:**
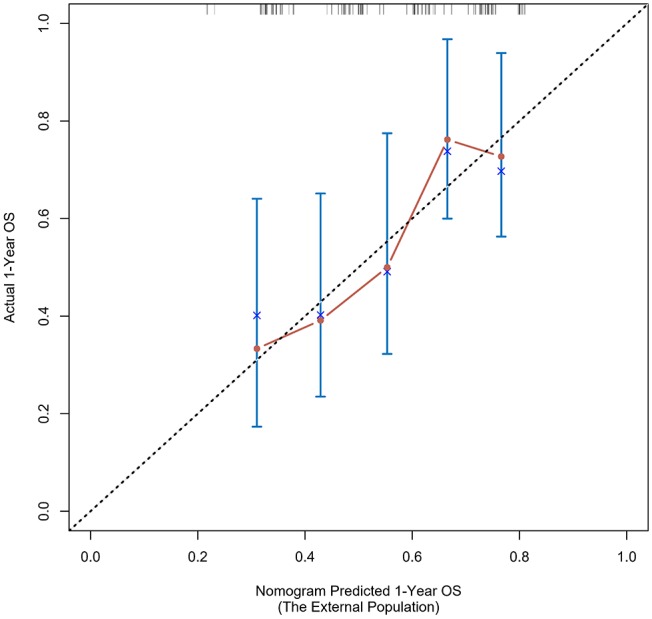
Calibration curves of the prognostic nomogram for 1-year overall survival in the external population.

**Figure 10:**
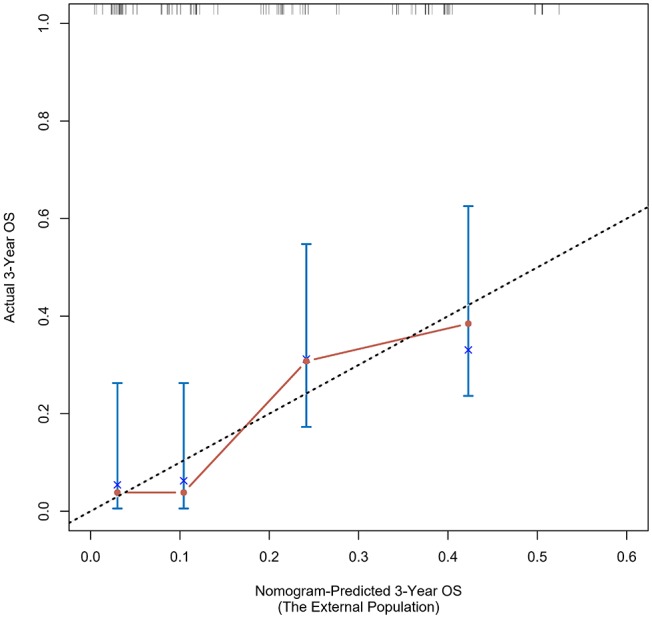
Calibration curves of the prognostic nomogram for 3-year overall survival in the external population.

**Figure 11:**
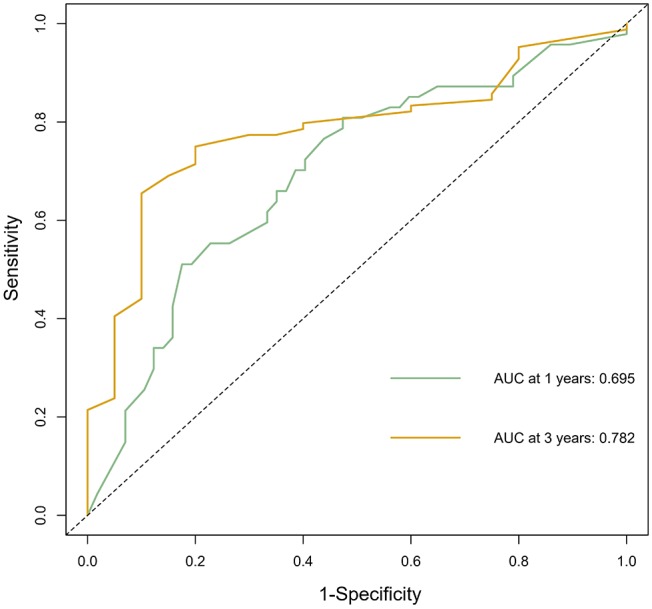
The ROC curve of the prognostic nomogram in the external population.

According to the nomogram and the score of our study, we drew the 1-year (Figure 9) and 3-year (Figure 10) calibration curves and ROC curve (Figure 11) of the external population, and the results of the curve have high consistency with those of our training group.

## Discussion

Due to limitations of diagnostic techniques, early-stage patients are often difficult to detect, resulting in poor prognosis of PDAC, so pancreatectomy is considered the main treatment currently, but its 5-year survival rate is low. Therefore, many researchers have done a lot of investigations for the improvement of the prognosis of PDAC. Many researches have shown that elevated markers may be associated with prognosis in patients with PDAC. Many prognostic factors have been defined, such as lymph node metastasis, tumor size, degree of differentiation, TNM stage, and vascular invasion. However, because these prognostic factors are difficult to determine before operation, scientists have made extensive research on prognostic serum markers in recent years. This study attempted firstly to establish prognostic nomogram combining serum markers (including inflammatory markers and tumor markers) and clinicopathological characteristics to assess the probability of 1-, 3-, and 5-year OS and to make a highly accurate model of PDAC patients.

Based on multivariate analysis, the results showed that TNM stage, CA199, CEA, globulin, NLR, and LMR were independent prognostic factors for OS. So we developed a nomogram of these markers, and the C-index was 0.697, which indicated our new model is highly accurate in predicting the prognosis of PDAC. Moreover, the AUC of the nomogram is larger than the AUC of other independent factors. Therefore, the nomogram based on multiple factors has greater prognostic value of PDAC patients.

In recent years, nomograms have shown high reliability for predicting tumor progression as a statistic model. Nomograms have better value for predicting prognosis than does TNM stage in some cancers (13, 14). This model has been identified as a new standard, and our study has the same conclusion that the AUC of a nomogram is larger than the AUC of TNM stage. Moreover, it can be applied in the clinic, which can help surgeons to evaluate the prognosis of patients and apply the appropriate treatment. As for PDAC patients, we can collect their clinical information and know their prognosis by their corresponding scores. As for patients with high scores, they need more to be further investigated through physical examination and follow-up, and clinicians need to conduct a comprehensive assessment to improve their prognosis. For surgeons and patients, this score system can help effectively.

Our nomogram contains six variables in which NLR and LMR are consistent with previous studies (15–18). Studies have suggested that systemic inflammation is an important factor which can affect the progression and long-term survival of cancer patients (19). As simple and inexpensive clinical markers, NLR, LMR, and PLR can reflect the state of inflammation, and they are associated with poor prognosis of some tumors but are less reported in PDAC. In this study, NLR and LMR were independent risk factors, while PLR was not. The possible mechanism is that the systemic inflammation caused by malignant tumors can releases a large number of pro-inflammatory mediators, such as CRP, fibrinogen, VEGF, and TGF-α. These factors stimulate tumor growth and metastasis (20); meanwhile, the antitumor immune response of T cells and natural killer cells in the system, which may be surrounded by a number of neutrophils, may decrease the opportunity to have contact with tumor cells and may have adverse effects on the prognosis of patients (21); besides, monocytes also play an important role in tumor progression (22). Macrophages (TAMs), which are derived from circulating monocytes, can promote tumor migration and proliferation (23). The study of Alexandros Giakoustidis also got the same conclusion (24), so NLR and LMR should be included in the regular assessment indexes of PDAC patients.

Globulin has attracted more and more researchers' attention as an independent prognostic indicator of tumor-related diseases in recent years. Albumin-to-globulin ratio was related with the prognosis of hepatocellular carcinoma, small-cell lung cancer, and nasopharyngeal carcinoma (25, 26). In the present study, our results suggest that globulin is significantly associated with the prognosis of PDAC patients. Globulin is a significant component of the inflammatory microenvironment and is produced by immune organs, including a large number of inflammatory response proteins. The level of globulin is related to the inflammatory status of the body. Besides, when the expression of globulin is too high, it can lead to damage of cells and suppress the immunologic function, which can contribute to the progression of malignant tumors (27). In addition to being a diagnostic marker for disease, globulin levels also play a key role in tumor progression and invasion in relation with cell proliferation and apoptosis (28–30).

Our research has several potential limitations: first, the external population needs more cases to verify the results; second, the included patients who had undergone surgical resection could not represent all PDAC patients.

In summary, TNM stage, CEA, CA199, globulin, NLR, and LMR levels were risk factors for the prognosis, and the novel nomogram model had reliable prognostic value for PDAC patients.

## Data Availability Statement

The data used to support the findings of this study are available from the corresponding author upon request.

## Ethics Statement

The studies involving human participants were reviewed and approved by the Ethics Committee of the First Affiliated Hospital of Anhui Medical University and the First Affiliated Hospital of Wenzhou Medical University. The patients/participants provided their written informed consent to participate in this study. Written informed consent was obtained from the individual(s) for the publication of any potentially identifiable images or data included in this article.

## Author Contributions

LZ and A-MX made contributions to the main work. They designed this study and drafted and revised this manuscript. LC provided and helped analyzed the data. All authors read and approved the final manuscript.

## Conflict of Interest

The authors declare that the research was conducted in the absence of any commercial or financial relationships that could be construed as a potential conflict of interest.

## Abbreviations

PDAC: pancreatic ductal adenocarcinoma
ROC curve: receiver operator characteristic curve
NLR: neutrophil-to-lymphocyte ratio
PLR: platelet-to-lymphocyte ratio
LMR: lymphocyte-to-monocyte ratio
AST: aspartate aminotransferase
ALT: alanine aminotransferase
GGT: the γ-glutamyl transpeptidase
C-index: concordance index
AUC: area under the ROC curve
OS: overall survival.
